# Coerced consent in clinical research: study protocol for a randomized controlled trial

**DOI:** 10.1186/s13063-024-08294-4

**Published:** 2024-07-04

**Authors:** Connor T. A. Brenna, Nancy Walton, Melanie Cohn, Urooj Siddiqui, Ella Huszti, Richard Brull

**Affiliations:** 1 Department of Anesthesiology & Pain Medicine, University of Toronto, Toronto, ON Canada; 2 Department of Physiology, University of Toronto, Toronto, ON Canada; 3 Yeates School of Graduate Studies, Toronto Metropolitan University, Toronto, ON Canada; 4 Krembil Brain Institute, University Health Network, Toronto, ON Canada; 5 Department of Anesthesiology and Pain Medicine, Mount Sinai Hospital, Toronto, ON Canada; 6 Biostatistics Department, University Health Network, Toronto, ON Canada; 7 Department of Anesthesia and Pain Management, University Health Network, Toronto, ON Canada; 8 Department of Anesthesia, Women’s College Hospital, Toronto, ON Canada

**Keywords:** Coercion, Voluntariness, Research consent, Clinical trials, Perioperative research

## Abstract

**Background:**

Despite the low-risk nature of participation in most clinical anesthesia trials, subject recruitment on the same day as surgery is often restricted due to the concerns of researchers and local research ethics boards that same-day consent may not afford adequate time and opportunity for patients to weigh and make decisions, as well as perceptions of patient vulnerability immediately prior to surgery that could impact the voluntary nature and the rigor of the informed consent process. However, specialties such as anesthesiology, critical care, interventional radiology, and emergency medicine have a varied pattern of practice and patient acquaintance that does not typically afford the luxury of time or, in many cases, advance consent for participation in research. Indeed, the initial encounter between anesthesiologists and patients undergoing elective procedures routinely occurs on the day of surgery. Concerns of coercion related to same-day consent for clinical anesthesia research trials have not been borne out in the literature, and represent a significant obstacle to clinical researchers, as well as to the patients who are denied opportunities for potential benefit through participation in research studies.

**Methods:**

We describe the protocol for a prospective randomized controlled trial examining the voluntariness of patient consent, solicited either in advance of surgery or on the same day, to participate in an anesthesia research study at Women’s College Hospital. One hundred fourteen patients scheduled to undergo ambulatory anterior cruciate ligament repair facilitated by general anesthesia with an adductor canal block will be randomized for recruitment either (a) in the pre-operative assessment clinic before the day of surgery or (b) on the day of surgery, to be approached for consent to participate in a fabricated research study of adjunct medications in adductor canal blocks. Regardless of allocation, patients in both groups will receive the same routine standard of care and will complete a post-operative questionnaire to signal perceptions of undue influence in the process of providing informed consent for the fabricated trial.

**Discussion:**

This study will inform trial design and practice guidelines surrounding the amount of time patients ought to be afforded in order to make durable decisions to participate (or not) in clinical research studies. This is expected to impact trial recruitment in a variety of clinical settings where researchers have only brief opportunities to interface with patients.

**Trial registration:**

The trial was registered prospectively on the Open Science Framework (OSF), registration #46twc, on 2023-Mar-17.

**Supplementary Information:**

The online version contains supplementary material available at 10.1186/s13063-024-08294-4.

## Administrative information

Note: the numbers in curly brackets in this protocol refer to SPIRIT checklist item numbers. The order of the items has been modified to group similar items (see http://www.equator-network.org/reporting-guidelines/spirit-2013-statement-defining-standard-protocol-items-for-clinical-trials/).

**Table:** 

Title {1}	Coerced consent in clinical research: study protocol for a randomized controlled trial
Trial registration {2a and 2b}.	Open Science Framework (OSF), registration #46twc.
Protocol version {3}	Date: 2024-Jun-18 (Version 1.1). Version 1.0 dated 2023-Dec-05.
Funding {4}	This trial is supported by the WCHAMSG Innovation Fund of the Alternative Funding Plan for the Academic Health Sciences Centres of Ontario.
Author details {5a}	Connor T. A. Brenna MD^1,2^, Nancy Walton RN BNSc PhD^3^, Melanie Cohn PhD CPsych^4^, Urooj Siddiqui MD FRCPC^5^, Ella Huszti PhD^6^, Richard Brull MD FRCPC^7,8^ ^1^ Department of Anesthesiology & Pain Medicine, University of Toronto, Toronto, ON, Canada ^2^ Department of Physiology, University of Toronto, Toronto, ON, Canada ^3^ Yeates School of Graduate Studies, Toronto Metropolitan University, Toronto, ON, Canada ^4^ Krembil Brain Institute, University Health Network, Toronto, ON, Canada ^5^ Department of Anesthesia, Mount Sinai Hospital, Toronto, ON, Canada ^6^ Biostatistics Research Unit, University Health Network, Toronto, ON, Canada ^7^ Department of Anesthesia and Pain Management, University Health Network, Toronto, ON, Canada ^8^ Department of Anesthesia, Women’s College Hospital, Toronto, ON, Canada
Name and contact information for the trial sponsor {5b}	Department of Anesthesia, Women’s College Hospital, 76 Grenville Street, Toronto, ON, M5S 1B2 Phone: (416) 323–6269 Fax: (416) 323–6307
Role of sponsor {5c}	The sponsor has played no part in the design of this trial, nor will it be involved in the collection, management, analysis, and interpretation of data, the writing of the report, or the decision to submit the report for publication.

## Introduction

### Background and rationale {6a}

Safeguarding patients’ interests and freedoms through a robust process of informed consent is a critical objective shared by clinicians, researchers, and research ethics boards (REBs). Notwithstanding the low-risk nature of participation in most clinical anesthesia trials, subject recruitment on the same day as surgery is often restricted due to the concerns of researchers and local REBs that same-day consent may not afford adequate time and opportunity for patients to weigh and make decisions [1], as well as perceptions of patient vulnerability immediately prior to surgery that could impact the voluntary nature and the rigor of the informed consent process. Researcher and REB concerns regarding same-day informed consent for participation in anesthesia research trials are theoretical and have not been robustly investigated in the current literature. Even though anxiety in the face of impending surgery is a normal human reaction, patients are still presumed to be capable of continuing to consent—or to revoke consent—to the actual surgery while they wait to enter the operating room. There is a paucity of evidence to suggest that a carefully conducted assessment of a patient’s capacity to understand information pertaining to a research trial, and to appreciate how a decision to participate would affect them, cannot be performed in this period. We suggest, in the absence of compelling evidence to the contrary, that patients are not so vulnerable during this period that they must be systematically protected by prohibiting any discussion of potential research participation in the immediate pre-operative period. Arguably, such a systematic prohibition is in fact ethically problematic in that, on its face, it can be viewed as a manifestation of paternalistic values which can deny patients the myriad potential benefits of research participation.

Chief among the roles and priorities of researchers and REBs alike is ensuring that the consent process is rigorous, and the autonomy of clinical research participants is respected. While the meaning of consent is both uniform and clear (that is, research participants must be capable of decision-making, fully informed, with ample time for consideration of options without undue influence, and their choice must be respected) [2], precisely what constitutes *ample time* resists clear definition. The World Health Organization states that “subjects must be given ample opportunity to enquire about the details of the trial […] sufficient time, determined by the patient’s health condition” [3]. The Tri-Council Policy Statement, representing Canadian standards for ethical research involving humans, declares that “for consent to be informed, prospective participants shall be given adequate time and opportunity to assimilate the information provided”, and that “the time required for this initial phase of the consent process will depend on such factors as the magnitude and probability of harms, the complexity of the information conveyed, and the setting where the information is given […]” [1]. In the United States, the American Medical Association is even less explicit, stating only that a valid consent process includes, “reviewing the process and any materials to ensure that it is understandable to the study population” [4]. Locally, the University of Toronto’s position on ‘ample time’ and suitability to consent is equally vague, only to consider “whether the contact person is known to the subject/authorized third party, has access to the patient information as part of their normal professional duties, or is able to assess capacity to consent” [5].

In the absence of uniform explicit recommendation or absolute quantification for what constitutes adequate time for patient reflection prior to consenting to participate in a clinical research trial [1, 3, 5], local REBs are left to develop their own stances on same-day consent practices, using a reasonable person standard and potential risks of harm in determining whether there must be a minimum lead time to facilitate patient contemplation. Although it has previously been argued that same-day research consent may be permissible [6], guidelines and recommendations for clinical research trial consenting practices vary widely. Table 1 summarizes available recommendations from international professional societies and government agencies regarding consent practices in the context of clinical research. Recommendations range from the oft-repeated requirement for “adequate” or “sufficient” time [7–9] to a more explicit demand for a lead time of at least 24 h to allow for patient consideration [10]. A notable deviation is the proportionate approach to seeking consent for clinical trials advised by the United Kingdom’s National Health Service (NHS) [11]. When seeking consent for patient participation in a clinical trial, the NHS recommends that “for research involving only minimal risks and/or little deviation from normal/standard clinical practice […] it may be reasonable to accept a decision taken at the time of approach” [11]. Additionally, aligning with sentiments expressed in the Canadian Tri-Council Policy Statement, the extent of information provided ought to be proportionate to the “nature and complexity of the research trial, risks, burdens and potential benefits, the ethical issues at stake” [11].

**Table 1:** International recommendations for same-day consent practices

Country	Institution	Guidelines/recommendations on timing of consent	Specifications for low-risk trials
Australia	National Health and Medical Research Council (2018) [7]	“Adequate time should be allowed for prospective participants to understand and consider what is proposed and for their questions and expression of concerns to be addressed by those obtaining their consent.”	“Proportionate to needs of participants, study risks and ethical sensitivity.”
Belgium	European Patient Forum (2016) [8]	“The patient must be given sufficient time to consider the decision.”	“A common problem for patients is that they are often given *too much information at once*… it does not contribute to their understanding … or help in balancing the risks and benefits involved. Thus, a more flexible and tailored approach should be applied that allows individual needs to be met.”
Denmark	National Videnskabsetisk Komité (2011) [10]	“The time for reflection depends on the nature of the trial. Basically, it should be at least 24 h.”	“The time for reflection depends on the *nature* of the trial.”
France	European Patient Forum (2016) [8]	“The patient must be given sufficient time to consider the decision.”	“A common problem for patients is that they are often given too much information at once … it does not contribute to their understanding … or help in balancing the risks and benefits involved. Thus, a more flexible and tailored approach should be applied that allows individual needs to be met.”
Ireland	Health Service Executive: Quality and Safety Patient Division (2022) [12]	“There are no legal provisions relating to the duration of consent for major interventions. However, it is good practice, where possible, to seek the person’s consent to the proposed intervention well in advance, so that there is sufficient time to respond to the person’s questions and provide adequate information.” “Consent for significant, sometimes major interventions such as an important surgical intervention should not be a once-off, ‘last minute’ event and should not be reduced to getting a hurried signature on a consent form. Accordingly, asking a person: • To provide consent just before an intervention is due to start, at a time when they may be feeling particularly vulnerable and unable to ask relevant questions; or • Seeking consent from someone who is sedated, in severe pain or extremely anxious creates doubt as to the validity of the consent. In particular, people should not be given routine pre-operative medication before being asked for their consent to proceed with a treatment.”	“Where the research entails only minimal risk, it is sufficient if the research offers the prospect of benefits either to the participants directly or to the group which is the focus of the research and to which the participants belong.” “Where the research poses more than minimal risk, it should aim to generate new knowledge of sufficient importance for addressing the participants’ conditions/needs. Such research should offer the prospect of direct benefits for the participants themselves and be commensurate with the level of foreseeable risk. The benefit-to-risk ratio presented by the research should be at least as favourable to participants as that presented by available alternative approaches..”
New Zealand	Auckland District Health Board (2018) [9]	“Sufficient time should be allowed for the patient to read the written information, and discuss this and any verbal information with whomever they wish.”	“The *higher the probability of risk* or the greater the magnitude of harm, the *more care and detail in giving information* is required.” “The patient must eb informed of rare risks that are more likely because of their particular circumstances, or which would have greater significance for that particular patient, e.g. the consequences of arm nerve damage for a carpenter.”
UK	NHS Health Research Authority (2019) [11]	“There are no definitive guidelines or legislation regarding the appropriate amount of time (or minimum amount of time) that potential participants should be allowed in order to consider whether to take part in research or not. A proportionate approach (in a non-urgent scenario) means that for more complex or burdensome studies a longer time may need to be provided for potential participants to consider their decision than that provided for simpler studies involving lower risks…For research involving only minimal risks…it may be reasonable to accept a decision taken at the time of approach.”	“A proportionate approach to seeking consent, i.e. adopting procedures commensurate with the balance of risk and benefits, should always be adopted so that potential participants are not overwhelmed by unnecessarily lengthy, complex and inaccessible information sheets but instead are provided with succinct, relevant, truthful information in a user- friendly manner that better promotes their autonomy.” “The methods and procedures used to seek informed consent and the level of information provided should be proportionate to: • Nature and complexity of the research • Risks, burdens and potential benefits • Ethical issues at stake”

#### Problems arising from required advance consent

Most physicians and surgeons meet with their patients on multiple occasions ahead of an intervention, affording these investigators time to identify, recruit, and enroll suitable research participants who are willing and able to provide informed consent. However, specialties such as anesthesiology, critical care, interventional radiology, and emergency medicine have a varied pattern of practice and patient acquaintance that does not typically afford the luxury of time or, in many cases, advance consent for participation in research [13]. Indeed, the initial encounter between anesthesiologists and patients undergoing elective procedures routinely occurs on the day of surgery. Recognizing our specialty’s unique practice patterns, the Canadian Anesthesiologists’ Society’s guidelines on the ethics of clinical research state that “pre-operative consent for clinical research in anesthesia may be obtained after admission to hospital, either before or on the day of the scheduled surgery” [14]. However, these guidelines are superseded by decisions of local REBs, and clinical investigators frequently face an impasse arising from their being positioned in a time-limited peri-operative system yet typically prohibited by REBs from consenting patients for clinical trials on the same day as their surgery [6, 13]. Conversely, clinicians participating in systems which do not discourage same-day consent may face moral distress in the absence of definitive evidence that such consent can be provided or procured without undue influence.

#### Current state of knowledge

Concerns of inadequate patient comprehension, time for contemplation, and privacy, as well as undue duress, coercion, and anxiety, continue to be cited as barriers to same-day consent for clinical anesthesia research trials [15]. These concerns, however, have not been adequately borne out in the literature. When consent is obtained on the same day as surgery, the vast majority of patients understand the intent of the clinical anesthesia trials, recognize that participation is voluntary, and appreciate that consent may be withdrawn at any time without consequence [16, 17]. Patients have been found capable of digesting consent form documents and making informed decisions about research participation in thirty minutes or less [16, 18]. Similarly, patients have reported that the peri-operative setting offers adequate privacy for consent discussions [16]. Purported coercion or undue influence of patients by their clinician investigators in the immediate pre-operative setting has also been refuted [16, 17]; one anesthesia study found that 97% of patients rated the pre-operative setting as “ideal” for obtaining informed consent to participate in clinical anesthesia trials [16]. The latter is most likely explained by patient preference for physicians with whom they will and/or must establish a relationship; accordingly, same-day consent provided in conversation with the responsible physician is likely superior to any surrogate [18]. Moreover, concerns of patient anxiety have not been realized as anesthesia researchers found no incremental increase in patient anxiety with same-day versus day-before recruitment and consent [19]. Finally, increasing the quantity of time for patient contemplation as a means to increase the quality of the informed consent process for anesthesia research has not been substantiated [20].

Similar to academic anesthesiologists, emergency medicine and radiology clinician-investigators have limited interaction with their potential trial participants, often meeting in a single encounter with no opportunity to discuss research recruitment and consent with their patients in advance of that encounter. Recognizing these limitations, REBs typically allow for deferred, targeted, or staged consent in order for patients to participate in emergency medicine clinical trials [21]. While such urgent or emergent adaptations to the standard informed consent process are not justified for the elective peri-operative setting wherein most anesthesia clinical trials occur, the same is not true for the radiology research experience. Indeed, low-risk radiology studies are generally approved for enrollment, recruitment, and consent on the same day as the radiological investigation or intervention [22]. The radiology (“X-ray”) department may not be equivalent to the operating room environment with respect to heightened patient anxiety; nonetheless, parallels are readily drawn between these two settings, including limited time and privacy, the potential for undue influence, and the low-risk nature of many radiology and clinical anesthesia trials.

#### Existing solutions

In many centers, the anesthesia pre-operative assessment clinic (PAC) has long served as the main permissible and fertile ground for subject recruitment to clinical research, presumably ensuring fully informed consent to participate in a clinical trial in the absence of any undue duress, and facilitating the establishment of a mutually trustful relationship. Principally purposed to mitigate or optimize patient-related factors that may increase risk of peri-operative complications, anesthesia PACs also typically function as the sole permissible venue (by our local REBs) for participant recruitment by research staff into clinical anesthesia research trials wherein subjects can provide informed consent days or weeks ahead of surgery. Unfortunately, however, this long-standing workaround presents unique obstacles to the appropriate recruitment of trial participants, in that the subset of patients referred for assessment in PACs tend to harbor a greater number and severity of comorbid conditions and are thus more likely to be ineligible for trial inclusion than those fitter patients who do not attend PAC [13] and would be more likely eligible for clinical anesthesia research. While the idea of coordinating with surgical colleagues to have healthy patients referred to PAC for the purpose of trial recruitment may be convenient for investigators, when balanced against the costs for patients (including inconvenience and lost income), the otherwise unnecessary use of hospital resources, health-care dollars, and PAC time constraints, the idea quickly loses appeal [16]. Moreover, following the COVID-19-related suspension of in-person assessments in anesthesia PACs across most academic centers, a large proportion of pre-operative assessments presently occur via telephone, presenting a major obstacle to trial recruitment and bringing to the fore longstanding sources of tension presented by same-day informed consent for clinical trials. While telephone calls solely for research recruitment have previously been used to introduce research protocols and initiate the informed consent process, many institutions consider these calls a violation of patient privacy as research personnel callers are not yet within the patient’s circle of care [13]. Furthermore, scheduling of calls, anxiety provoked from unsolicited calls originating from the hospital, and constraints in time and human resources represent important ethical and logistical challenges [18, 23].

Conceivably, the COVID-19 pandemic has altered patient and provider views on telephone or videoconference as means to identify, recruit, enroll, and consent for research protocols. Though the pandemic has already rendered telemedicine more applicable and acceptable to patients and practitioners alike, whether or not it could or should penetrate clinical research programs to a similar degree, especially with respect to preserving the sanctity of privacy within the circle of care, will require further consideration [18, 24]. Ongoing requirements for universal masking inside of hospitals may further complicate recruitment and consent for clinical trials as clinician investigators must first establish a relationship founded on trust with potential research participants. While it removes the physical face-to-face component of a patient-physician interaction, one potential advantage of videoconferencing is that it does allow unencumbered facial recognition and mutual awareness of affect. Thus, the persisting effects of telemedicine on clinical research programs beyond this pandemic are yet unknown and warrant further study, including the patients’ understanding and appreciation of disclosed information, perceptions of the consent process, concepts of ample time for decision-making, patient perceptions of coercion and ability to make decisions voluntarily, and research recruitment rates. These workarounds aside, telemedicine presents several shortcomings with respect to clinical assessment, including the inability to perform a thorough physical exam, and we expect pre-operative assessments to return to an in-person PAC setting.

#### Significance

The validity of same-day consent for anesthesia research trials has been widely supported [6, 13, 16–19]. However, this phenomenon has not been robustly investigated. Prohibiting same-day consent for clinical anesthesia trials is an overly burdensome exercise for both clinical investigators and research staff and threatens the systematic exclusion of patients otherwise fit and competent who may benefit from participation in clinical anesthesia research trials. The proposed study will determine patients’ perceptions of voluntariness in the provision of informed consent at early and late timepoints prior to surgery and is expected to inform subsequent peri-operative trial design and guidelines pertaining to the amount of time patients ought to be afforded to make informed decisions to participate (or not) in research.

## Objectives {7}

We aim to determine whether a patient’s consent to participate in a low-risk clinical anesthesia research trial obtained on the same day as their surgery is as voluntary as consent obtained prior to the day of surgery. The voluntariness of consent will be determined based on the patient’s perceptions of undue influence using an adaptation of the Iowa Coercion Questionnaire [25] and Coercion Assessment Scale [26].

We hypothesize that patients from whom consent for trial participation is obtained on the same day as surgery will report similar voluntariness, as measured by perceptions of undue influence, as those in whom consent for participation was obtained prior to the day of surgery.

## Trial design {8}

**Fig. 1:**
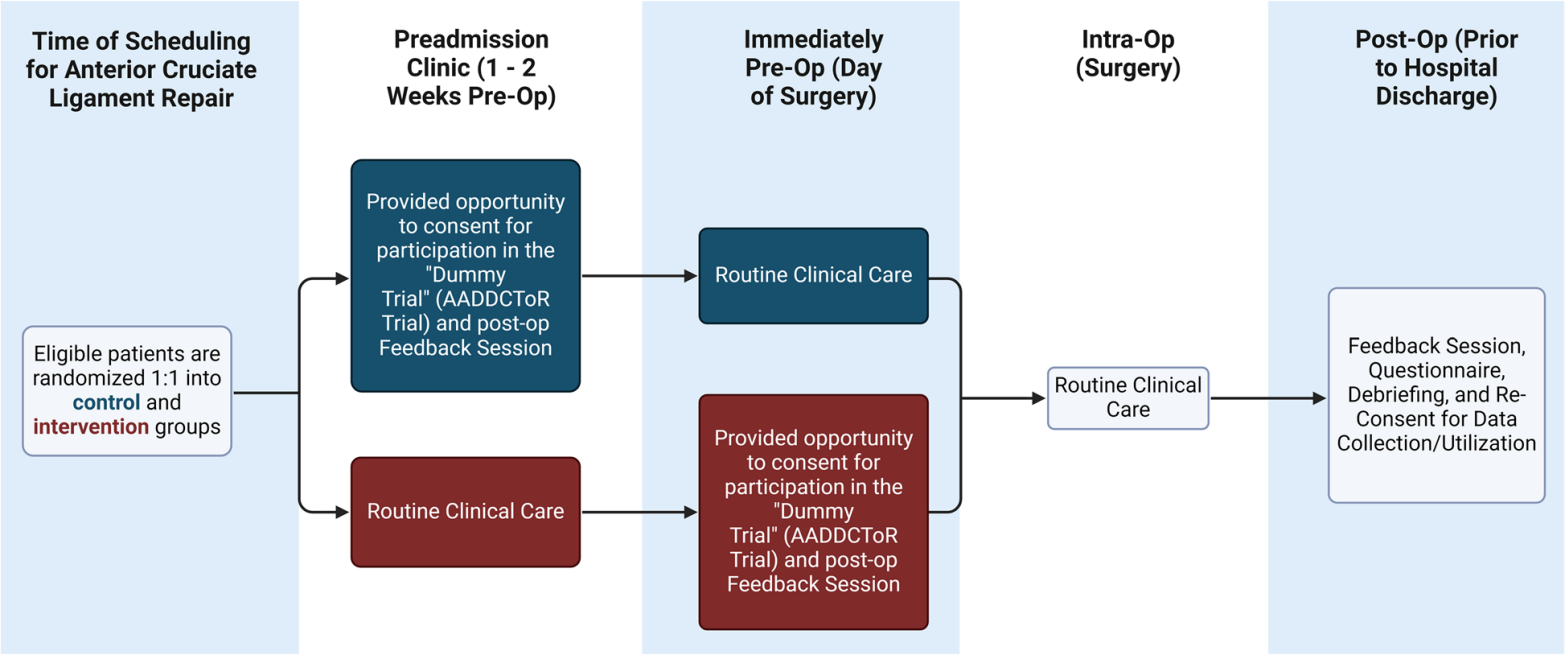
Study design flow diagram

Due to the present study’s focus on measuring undue influence, which may not be consciously perceived by patients and is uniquely vulnerable to bias, the described trial relies on an element of deception (similar to psychological studies of phenomena in which nondeceptive procedures are not feasible) [27]. We propose a prospective single-blinded randomized controlled non-inferiority trial (the ‘Consent Study’) examining the voluntariness of patient consent to participate in an anesthesia research trial (the ‘Dummy Trial’; a fictitious low-risk randomized clinical anesthesia research trial) at Women’s College Hospital (WCH) when such consent is obtained (a) prior to the day of surgery compared to (b) on the same day as surgery compared to. The study design is illustrated as a flow diagram (provided in Fig. 1), and presented here in the form of a SPIRIT Figure.

**Table:**
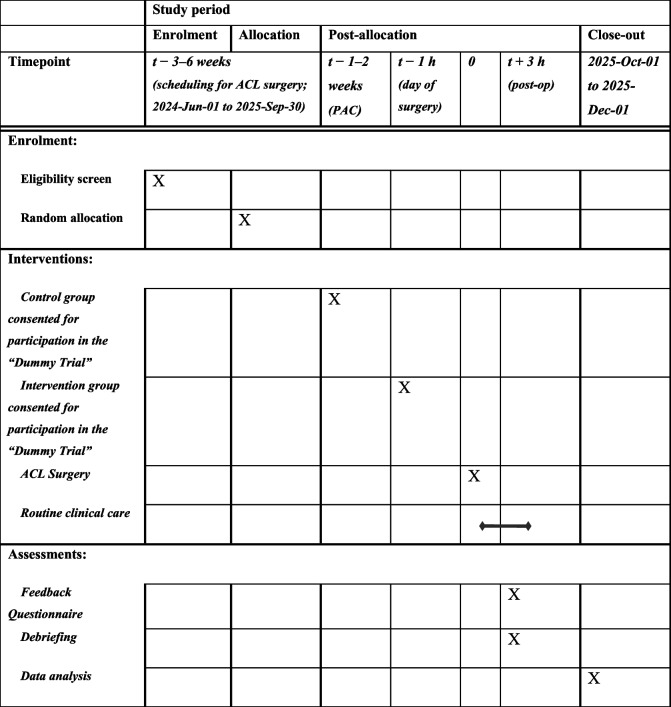


*ACL* anterior cruciate ligament, *PAC* preadmission clinic

For the purposes of the Consent Study, all patients will be recruited at one of these two timepoints to participate in the Dummy Trial, a fictitious low-risk randomized clinical anesthesia research trial entitled “The AADDCToR Trial: A Randomized-Controlled Comparison of the Analgesic Effects Following Anterior Cruciate Ligament Repair.”, for which a trial protocol has already been approved by our local REB (REB# 2018-0164-B, 2019-Mar-01) and registered [28], but not yet completed. The Dummy Trial purports to compare the efficacy of adjuncts (dexamethasone and/or dexmedetomidine) combined with local anesthetics in adductor canal blocks for post-operative analgesia.

To preserve the deception required for naturalistic observation, the present trial was pre-registered in advance of participant recruitment and published under an embargo to prevent eligible participants from prematurely unblinding themselves to the true nature of the study.

## Methods: participants, interventions, and outcomes

### Study setting {9}

This is a single-center study undertaken at WCH, an academic hospital in Toronto, Canada.

### Eligibility criteria {10}

Patients scheduled to undergo ambulatory anterior cruciate ligament (ACL) repair facilitated by general anesthetic with an adductor canal block (the anesthetic standard of care for this operation) at WCH will be eligible to participate in the Consent Study. As per our standard practice, all patients undergoing ACL repair will be assessed 1–2 weeks prior in our PAC. The specific eligibility criteria will be:

Inclusion criteria

- Presentation at the WCH PAC for outpatient ACL repair surgery with general anesthesia and an adductor canal block.

Exclusion criteria

- Inability to provide informed consent.
  - ◦ All patients seen in the PAC and on the surgical date for this elective procedure have provided informed consent to undergo their surgery, and so it is presumed that they will have capacity to consent to the present study without former assessment. However, any patient in which there is an indication that they cannot consent for themselves in the surgical consent process would not be approached for this study.

### Who will take informed consent? {26a}

Eligible participants will be approached by a clinical research coordinator with an invitation to participate in the Dummy Trial. Recruitment will follow standard processes, using an informed consent package identical to that devised for the AADDCToR Trial (with only the dates and investigator information updated).

Additionally, in order to measure the extent of voluntariness related to the Dummy Trial consent process, all patients recruited at either timepoint will be asked to consent to completing a concise questionnaire and debrief relating to their peri-operative experience (“Feedback Session”), after their surgery. Because the nature of the Consent Study will be explained in the Feedback Session, patients will not be offered an opportunity to consent to only the Dummy Trial and not the Feedback Session. Any patients who choose not to provide consent for participation in the Dummy Trial and Feedback Session will receive the standard of care (identical to those who consent for participation). However, because we believe that patients who elect not to consent to the Dummy Trial may be able to offer critical insights about perceptions of coercion or undue influence in invitations to participate in clinical research, these patients will be approached once more in the post-operative setting before discharge from hospital with a final invitation to participate in the Feedback Session.

### Additional consent provisions for collection and use of participant data and biological specimens {26b}

On the consent form, participants will be asked if they agree to the use of their data should they choose to withdraw from the trial. They will also have the opportunity to reaffirm their consent for this data to be analyzed, following the post-operative Feedback Session. Furthermore, participants will be asked to grant permission for the research team to share relevant data with people from the University taking part in this research and/or from regulatory authorities, where relevant. This trial does not involve collecting biological specimens for storage.

## Interventions

### Explanation for the choice of comparators {6b}

Patients randomized to the control group will be approached and invited to participate in the Dummy Trial at the time of their PAC visit. As per our standard practice for peri-operative trial recruitment, informed consent for participation in the Dummy Trial will be obtained from patients at the time of their PAC visit prior to the day of surgery.

### Intervention description {11a}

Patients randomized to the intervention group will complete their PAC visit without recruitment to any research trials. Patients in this group will be approached and invited to participate in the Dummy Trial pre-operatively, on the same day as surgery.

The Dummy Trial purports to compare the efficacy of adjuncts (dexamethasone and/or dexmedetomidine) combined with local anesthetics in adductor canal blocks for post-operative analgesia. However, all patients will receive the standard of care (an adductor canal block placed in a proximal location followed by a general anesthetic) with no true comparison of nerve block adjunct efficacy, regardless of their decision to participate in the Dummy Trial.

The Feedback Session will be an individualized session conducted post-operatively for all patients who consent to participate in the Dummy Trial, prior to discharge from hospital. The Feedback Session will be comprised of a pen-and-paper written questionnaire (modified by our research team from the Iowa Coercion Questionnaire and Coercion Assessment Scale) [25, 26]. This questionnaire comprises demographic information and items probing participants’ perception of the consent process which they undertook, and is provided as Additional file 1. If any specific accommodation or assistance is required to complete the pen-and-paper questionnaire, our study team will strive to provide these. The written questionnaire, which we anticipate will take between 5 and 10 min, will be followed by a scripted 10-min verbal debrief prior to discharge from hospital on the day of surgery, which serves to inform participants of the true nature and intent of the Consent Study and Dummy Trial. At this time, patients will have an opportunity to reconfirm consent, in keeping with Article 3.7A of the TCPS [1]. Patients who initially declined to participate in the Dummy Trial, but agree to participate in the Feedback Session, will be provided the same questionnaire and verbal debrief to assess their perceptions of the consent process and explain the intention of the study they had been invited to participate in.

### Criteria for discontinuing or modifying allocated interventions {11b}

Not applicable: this trial does not account for the discontinuation or modification of allocated interventions, as allocation precedes patient recruitment.

### Strategies to improve adherence to interventions {11c}

Not applicable: adherence to interventions is not dependent on participants, in the context of this trial.

### Relevant concomitant care permitted or prohibited during the trial {11d}

All participants in this trial, regardless of group allocation, will receive the standard of care.

### Provisions for post-trial care {30}

No harms are anticipated to arise in the course of this trial. In order to minimize the possibility of patient distress (e.g., after revealing the deceptive nature of the Dummy Trial), our debriefing meetings will contain a thorough explanation of the rationale for deception to measure the psychological construct of voluntariness in the Consent Study. Nevertheless, our debriefing team will actively monitor for any adverse events such as patient distress, and inquire about patients’ emotional state at the end of the debriefing session. Any acutely distressed patient will be referred to or directly provided support services such as counseling; several members of our study team are professionally trained to manage distress and provide emotional support should this need arise. Any instances of patient distress will be carefully documented as an adverse event, and included in the study team’s regular review. This information will be used to assess whether any trial protocol adjustments can be made to minimize discomfort without compromising scientific integrity, or whether prematurely stopping the study should be considered.

### Outcomes {12}

The primary outcome is participants’ score on the post-operative questionnaire, which is designed to reflect perceptions of undue influence to consent to participation in the Dummy Trial.

Secondary outcomes include patients’ perceptions of how informed they were with respect to the consent provided to participate in the Dummy Trial, and rate of enrollment in the Dummy Trial. In addition, an exploratory secondary analysis is planned to examine the potential relationships between participants’ demographic variables and the primary outcome.

### Participant timeline {13}

Participants will be randomized into groups invited to participate in the Dummy Trial either in the PAC (approximately 1–2 weeks in advance of surgery) or on the day of surgery. Post-operatively, prior to discharge from hospital, they will be approached to complete the Feedback Session including completion of the study questionnaire and debriefing. A flow diagram illustrating the study’s design is provided in Fig. 1. There will be no later follow-up assessments.

### Sample size {14}

Based on our hypothesis that voluntariness will be similar between the two study groups, this trial is powered as a non-inferiority RCT. Drawing on data from previous reports validating the questionnaires adapted for this trial [26], we calculate that a sample size of 102 patients (51 in each group) will be needed to assess that the intervention group is non-inferior to the control group with 80% power (alpha = 0.05), with respect to the main outcome defined as the average questionnaire score (continuous measure ranging from 1 to 4), using a two-sample equal-variance *t*-test. We assumed a non-inferiority margin of − 0.3 and a common standard deviation of 0.6 (assumed to be a 5th of the average score range). In order to account for the assumed at most 10% of patients who would decline to participate in this Feedback Session, having not enrolled in the Dummy Trial, the randomization sample size will be 114 patients (57 in each group). The sample size was computed using PASS 2023, version 23.0.2.

### Recruitment {15}

Recruitment will account for the possibility of study drop-out, as described above, and will follow identical protocols to those commonly used for peri-operative trials in our center (using the AADDCToR Trial recruitment model as a template).

## Assignment of interventions: allocation

### Sequence generation {16a}

At the time of their PAC visit, all study-eligible patients will be randomized to one of two groups by a computer-generated sequence. This will be done using a 1:1 block-randomized with varying block sizes (from 2 to 6).

### Concealment mechanism {16b}

Study participants will not be aware of their group allocation, which will be securely cataloged in a REDCap database, until after data lock. Randomization will be performed within REDCap using a program to pull numbers from the computer-generated sequence and assign them to patients at the time of recruitment; there is therefore no human involvement, and the process is fully concealed from both prospective participants and (until the study arm is assigned) prospective investigators. Further concealment measures include trial pre-registration with an embargo so that eligible participants are not able to prematurely unblind themselves to the true nature of the study. Balancing this with the transparency afforded by protocol publication, this protocol was submitted for publication shortly before participant recruitment began to afford a maximum lead time before records of the Consent Study would be made publicly accessible. Data will be provided by these blinded participants through the questionnaire, rather than collected by study personnel.

### Implementation {16c}

The principal author will generate the allocation sequence for the trial within the REDCap database. A clinical research associate will enroll participants on the basis of this allocation.

## Assignment of interventions: blinding

### Who will be blinded {17a}

Trial participants and care providers will be blinded to group assignment. Trial data will be given by participants and, after data lock, data analysts will be unblinded to grouping to compare questionnaire scores between groups.

### Procedure for unblinding if needed {17b}

Not applicable: given the fictitious nature of the Dummy Trial, there is no procedure in place for unblinding of group allocation.

## Data collection and management

### Plans for assessment and collection of outcomes {18a}

The primary study instrument (Feedback Session questionnaire) is provided in Additional file 1. It is derived, for greater fidelity to the peri-operative patient population, from two validated tools for the measurement of undue influence: the Iowa Coercion Questionnaire [25] and Coercion Assessment Scale [26].

### Plans to promote participant retention and complete follow-up {18b}

Not applicable: the trial period is extremely brief, and we do not anticipate difficulty with participant retention or completion of follow-up.

### Data management {19}

All information gathered during the course of the study will anonymized and stored in a secure, locked file cabinet. Only research personnel will have access to the cabinet key. Survey data will be extracted by a study team member blinded to group allocations into a secure REDCap database managed by the trial steering committee.

### Confidentiality {27}

Patient numbers will be used instead of names to ensure confidentiality upon entry and analysis of data. When results are prepared for presentation or publication, they will be presented in a way that makes it impossible to identify individual participants.

### Plans for collection, laboratory evaluation, and storage of biological specimens for genetic or molecular analysis in this trial/future use {33}

Not applicable: biological specimens will not be collected in the described trial.

## Statistical methods

### Statistical methods for primary and secondary outcomes {20a}

We will use descriptive statistics to summarize demographic information and baseline characteristics. We will reorder item responses from least to greatest perception of coercion (e.g., “someone tried to force me to be in this study” would be unchanged, with 1 = “not at all”, while “I chose to be in this study” would flip such that 1 represents “very much so) to calculate overall undue influence scores for each discrete section and the questionnaire as a whole. We will perform chi-square tests for ordinal responses to each item on the study questionnaires. We will also define the total average score for each questionnaire as the sum of individual item answers divided by the total questions answered, resulting in a continuous measure ranging from 1 to 4. To compare these average scores in patients approached for research consent prior to or on the day of surgery, we will use t-tests or non-parametric Wilcoxon tests.

### Interim analyses {21b}

Not applicable: no interim analyses are planned in this trial.

### Methods for additional analyses (e.g., subgroup analyses) {20b}

As a secondary analysis, we will use univariable and multivariable linear regression models to test the potential associations of collected demographic factors with the average questionnaire scores.

### Methods in analysis to handle protocol non-adherence and any statistical methods to handle missing data {20c}

Average questionnaire scores will be calculated by dividing the sum of scores of completed questions by the number of completed questions, thereby accounting for missing questionnaire data. No allotments are made for the management of protocol non-adherence.

### Plans to give access to the full protocol, participant-level data, and statistical code {31c}

The anonymized datasets analyzed during the current study, as well as the statistical code, are available from the corresponding author upon reasonable request, as is the full protocol.

## Oversight and monitoring

### Composition of the coordinating center and trial steering committee {5d}

The trial steering committee comprises various areas of content expertise, including clinical anesthesia, neuropsychology, and research ethics. It is composed of three physicians, a research ethics scientist, a clinical neuropsychologist, and a statistician. This group will meet on a monthly basis as well as ad hoc to provide oversight to the trial, review adverse events, and fulfill reporting obligations. A second group comprising two physicians, a research coordinator, and several research assistants will be responsible for the day-to-day management of the trial (e.g., patient recruitment, assessment, and debriefing) and will meet on a weekly basis as well as ad hoc.

### Composition of the data monitoring committee, its role and reporting structure {21a}

Not applicable: data will be reviewed by two independent investigators, but no protocol changes will be made on the basis of any interim analysis and no data monitoring committee has been established.

### Adverse event reporting and harms {22}

Not applicable: as this trial does not involve any physical intervention, no mechanism for reporting adverse events or harms has been established.

### Frequency and plans for auditing trial conduct {23}

A data monitoring committee was not considered as the trial examines a low-risk intervention. However, the trial steering committee will meet on a monthly basis to review trial conduct throughout the trial period.

### Plans for communicating important protocol amendments to relevant parties (e.g. trial participants, ethical committees) {25}

Any amendments to the trial protocol, conduct, or management will be submitted to the appropriate institution REBs and receive the necessary approvals prior to implementation.

### Dissemination plans {31a}

A dissemination plan will be produced by the trial steering committee following data analysis. The anticipated outputs will include scientific presentation and openly accessible academic papers in leading peer-reviewed journals.

## Discussion

Not applicable: the protocol of this trial addresses several unique practical and operational issues inherent in the deceptive nature of the intervention, in other sections.

## Trial status

The trial protocol was registered on 2023-Mar-17 (version 1.0). Patient recruitment is expected to begin on 2024-Jun-01, and estimated to end on 2025-Sep-30.

### Supplementary Information

Additional file 1: Appendix I. Feedback Session Questionnaire 

## Data Availability

All contributing authors will have access to the anonymized final trial dataset.

## Abbreviations

REB: Research Ethics Board
PAC: Pre-operative assessment clinic
