# Supplementary material for: Coerced consent in clinical research: study protocol for a randomized controlled trial
Source: Trials. 2024 Jul 4;25:452. doi: 10.1186/s13063-024-08294-4 (PMC11223386; doi:10.1186/s13063-024-08294-4)
Supplement: Supplementary file 1 — Additional file 1: Appendix I. Feedback Session Questionnaire [file 13063_2024_8294_MOESM1_ESM.docx]

**Appendix I: Feedback Session Questionnaire**

**SECTION A: Demographics**

**We are interested in gathering a bit of information about you. We will not be asking for your name, so your name will not be connected to your responses. By providing information about demographic characteristics it helps us to make comparisons with previous research in the area.**

**A1. Today’s date: _____­­­­­__________**

**A2. Your age: _____**

**A3. Sex:**

🞏 Male

🞏 Female

🞏 Prefer to specify: _________

🞏 Prefer not to specify

**A4. Gender:**

🞏 Man

🞏 Woman

🞏 Prefer to specify: _________

🞏 Prefer not to specify

**A5. a) Prior to age 16, were you raised predominately in Canada (IF YES, SKIP to A6)?**

🞏 Yes

🞏 No

🞏 Prefer not to respond

**b) If not, where were you predominately raised prior to age 16?**

🞏 United States

🞏 Mexico

🞏 South America, Central America, or the Caribbean

🞏 Europe

🞏 Africa

🞏 Asia

**c) How many years have you lived in Canada? _____**

**d) How many years of your education were primarily in English? ______**

**A6. Do you identify as belonging to any minority groups (e.g., ethnic, racial, religious, sexual orientation, etc)?**

🞏 Yes, the following visible minorities: _____________

🞏 Yes, the following invisible minorities: _____________

🞏 No

🞏 Prefer not to say

**A7. Do you experience any disabilities or exceptionalities (e.g., sensory, physical, intellectual, etc)?**

🞏 Yes, the following visible disabilities/exceptionalities: _____________

🞏 Yes, the following invisible disabilities/exceptionalities: _____________

🞏 No

🞏 Prefer not to say

**A8. Marital Status:**

🞏 Married or living with someone in marital-like relationship

🞏 Never married & never lived with someone in a marital-like relationship

🞏 Separated

🞏 Divorced or formerly lived with someone in a marital-like relationship

🞏 Widowed

**A9. How many years of education have you completed in each of the following areas?**

**Grade school:** 🞏0 🞏1 🞏2 🞏3 🞏4 🞏5 🞏6 🞏7 🞏8 🞏9 🞏10 🞏11 🞏12 🞏13

**Apprenticeship program:** 🞏0 🞏1 🞏2 🞏3 🞏4 🞏5 🞏6

**College/Technical/Business/Vocational/Nursing (non-university):**

🞏0 🞏1 🞏2 🞏3 🞏4 🞏5 🞏6

**University program:** 🞏0 🞏1 🞏2 🞏3 🞏4 🞏5 🞏6 🞏7 🞏8 🞏9 🞏10 or more

**Research-based training (e.g., graduate studies):** 🞏0 🞏1 🞏2 🞏3 🞏4

**Health-related profession:** 🞏0 🞏1 🞏2 🞏3 🞏4 🞏5 🞏6

**SECTION B: Informed Decision Making**

**We are interested in your opinion on how informed you felt prior to entering this study. Please rate each item on the 4-point scale provided.**

**B1. I felt like I had enough time to read and understand the consent form.**

1 2 3 4

(Not at all) (Somewhat) (Moderately So) (Very much so)

**B2. I felt like I understood the purpose of the study.**

1 2 3 4

(Not at all) (Somewhat) (Moderately So) (Very much so)

**B3. I felt like I had enough time to make a decision about whether or not to participate.**

1 2 3 4

(Not at all) (Somewhat) (Moderately So) (Very much so)

**B4. I felt like my questions were adequately answered.**

1 2 3 4

(Not at all) (Somewhat) (Moderately So) (Very much so)

**B5. I felt anxious about participating in this study.**

1 2 3 4

(Not at all) (Somewhat) (Moderately So) (Very much so)

**B6. My anxiety made it difficult for me to pay attention to the study description.**

1 2 3 4

(Not at all) (Somewhat) (Moderately So) (Very much so)

**B7. Have you ever participated in research before?**

🞏 Yes

🞏 No

**If YES, please answer the following. If NO, please skip to Section C.**

**B8. I feel comfortable participating in research.**

1 2 3 4

(Not at all) (Somewhat) (Moderately So) (Very much so)

**B9. I understand the risks and benefits of participating in research.**

1 2 3 4

(Not at all) (Somewhat) (Moderately So) (Very much so)

**B10. I worry about the impact of research on my medical care/treatment.**

1 2 3 4

(Not at all) (Somewhat) (Moderately So) (Very much so)

**SECTION C: Pressure to participate**

**We are also interested in your opinion on whether you felt pressured to participate in this study. Please rate each item on the 4-point scale provided.**

**C1. I felt free to do what I wanted when deciding whether or not to be in this study.**

1 2 3 4

(Not at all) (Somewhat) (Moderately So) (Very much so)

**C2. Someone tried to force me to be in this study.**

1 2 3 4

(Not at all) (Somewhat) (Moderately So) (Very much so)

**C3. I chose to be in this study.**

1 2 3 4

(Not at all) (Somewhat) (Moderately So) (Very much so)

**C4. I had control over whether or not I was in this study.**

1 2 3 4

(Not at all) (Somewhat) (Moderately So) (Very much so)

**C5. I made the decision to participate in this study.**

1 2 3 4

(Not at all) (Somewhat) (Moderately So) (Very much so)

**C6. I felt like entering the study would improve my medical care.**

1 2 3 4

(Not at all) (Somewhat) (Moderately So) (Very much so)

**C7. I thought I might be punished if I didn’t participate.**

1 2 3 4

(Not at all) (Somewhat) (Moderately So) (Very much so)

**C8. I thought it would look bad to my doctor if I did not enter the study.**

1 2 3 4

(Not at all) (Somewhat) (Moderately So) (Very much so)

**C9. I thought the doctor would like it if I entered the study.**

1 2 3 4

(Not at all) (Somewhat) (Moderately So) (Very much so)

**C10. I entered the study even though I did not want to.**

1 2 3 4

(Not at all) (Somewhat) (Moderately So) (Very much so)

**C11. It would have created problems between me and the medical team if I did not participate.**

1 2 3 4

(Not at all) (Somewhat) (Moderately So) (Very much so)

**C12. I participated to avoid being bored.**

1 2 3 4

(Not at all) (Somewhat) (Moderately So) (Very much so)

**C13. I participated because it could help researchers learn new information to help others.**

1 2 3 4

(Not at all) (Somewhat) (Moderately So) (Very much so)
